# Anti-Oxidant and Tyrosinase Inhibitory In Vitro Activity of Amino Acids and Small Peptides: New Hints for the Multifaceted Treatment of Neurologic and Metabolic Disfunctions

**DOI:** 10.3390/antiox8010007

**Published:** 2018-12-26

**Authors:** Grazia Luisi, Azzurra Stefanucci, Gokhan Zengin, Marilisa Pia Dimmito, Adriano Mollica

**Affiliations:** 1 Department of Pharmacy, G. d’Annunzio University of Chieti-Pescara, 66100 Chieti, Italy; gluisi@unich.it (G.L.); a.stefanucci@unich.it (A.S.); marilisa.dimmito@unich.it (M.P.D.); 2 Department of Biology, Science Faculty, Selcuk University, Konya 42130, Turkey; Gokhagokhanzengin@selcuk.edu.tr

**Keywords:** anti-oxidant, scavenger, neurodegenerative diseases, metabolic disorders

## Abstract

Oxidative damage is among the factors associated with the onset of chronic pathologies, such as neurodegenerative and metabolic diseases. Several classes of anti-oxidant compounds have been suggested as having a protective role against cellular stressors, but, in this perspective, peptides’ world represents a poorly explored source. In the present study, the free radical scavenging properties, the metal ion reducing power, and the metal chelating activity of a series of sulfurated amino acids and tripeptides were determined in vitro through canonical assays (DPPH, ABTS, CUPRAC, FRAP, PM, and EECC) and estimated in comparison with the corresponding activities of synthetic peptide semicarbazones, incorporating the peculiar non-proteinogenic amino acid, *tert*-leucine (*t*Leu). The compounds exhibited remarkable anti-oxidant properties. As expected, sulfurated compounds **1**–**5** were found to be the most efficient radical scavengers and strongest reductants. Nevertheless, *t*Leu-containing peptides **7** and **8** disclosed notable metal reducing and chelating activities. These unprecedented results indicate that *t*Leu-featuring di- and tripeptide backbones, bearing the semicarbazone chelating moiety, are compatible with the emergence of an anti-oxidant potential. Additionally, when tested against a panel of enzymes usually targeted for therapeutic purposes in neurodegenerative and metabolic disorders, all samples were found to be good inhibitors of tyrosinase.

## 1. Introduction

Since the first introduction of the term, “oxidative stress” (OS), by Helmut Sies a few decades ago [1], an overwhelming body of biomedical literature has flourished on the topic [2,3,4,5]. The original concept of the phenomenon refers to an undesired production of pro-oxidant species, such as oxygen (ROS) and nitrogen (RNS) free radicals, that are not adequately counterbalanced neither by the anti-oxidant defense mechanisms of the organism, nor by the supply of natural anti-oxidants, finally resulting in severe damage of lipids, proteins, and DNA. The OS meaning has now been updated to acknowledge free radicals positive role as redox-signaling molecules in healthy tissues of aerobic organisms [6,7]. Nevertheless, a plethora of studies has been reported on the claimed anti-oxidant properties of chemicals, foods, or plant components to underline the possible benefits against OS-related pathologies. It is otherwise well established that that the anti-oxidant mechanisms are based on the physiologic enzymatic and non-enzymatic redox buffering systems more than on nutritional supplements [8].

The overall anti-oxidant potential of a specific compound, whose chemical reactivity towards disparate toxic species may consistently differ, depends upon the variable contribution of features as the free radical scavenging capacity, the reducing and redox buffering effectiveness, and the metal-chelating properties. Accordingly, a range of specific anti-oxidant evaluation tests are available, which may be appropriately selected to gain information about the precise mechanism underlying a certain effect, and serve to characterize the anti-oxidant character in the whole, expressed as the total anti-oxidant capacity (TAC) of the compound. Since the TAC measures only part of the anti-oxidant power, usually excluding enzymatic activities, the non-enzymatic antioxidant capacity (NEAC) has been recently suggested as a more fitting term [9].

The radical scavenging property refers to the molecule ability to quench oxygen (HO, HOO, ROO, H_2_O_2_, O_2_^1^), nitrogen (NO, HOONO), and chlorine (HOCl) free radicals or radical generating species, thereby blocking radical chain reactions. Compounds of this type, also known as chain-breaking anti-oxidants, well apart from preventive anti-oxidants that inhibit the formation of reactive oxygen species, may act by two distinct mechanisms, which imply competition with biological substrates for the (*i*) hydrogen atom transfer (HAT), or the (*ii*) single electron transfer (SET) from radicals. In the majorities of assays for the HAT-based reactions, free radicals are thermally generated through the decomposition of azo-compounds. The oxygen radical absorbance capacity (ORAC) method, the total radical trapping antioxidant parameter (TRAP) test, and the lipid peroxidation assay (LPA) are commonly used. The assays for SET reactions compare the in vitro capacity of the anti-oxidant to reduce the oxidant cromophore in comparison with reference reactants as: Trolox, in the trolox equivalent antioxidant capacity (TEAC) test, 2,2′-azino-bis(3-ethylbenzothiazoline)-6-sulphonic acid (ABTS), in the ABTS radical scavenging efficacy assay, and 2,2-diphenyl-1-picrylhydrazyl (DPPH), in the DPPH analogous test. It should be borne in mind, however, that a precise boundary between these two mechanisms does not exist, so that ABTS and DPPH are commonly considered mixed-mode assays [10] (Table 1).

Reductant agents are electron-rich molecules, which exert their anti-oxidant effect by an electron-transfer mechanism. This character is determined through a panel of tests for the direct estimation of the reducing capacity of the substance, such as the ferric ion reducing antioxidant Power (FRAP), the cupric antioxidant capacity (CUPRAC) method, and the phosphomolybdenum (PM) assay. FRAP and CUPRAC tests measure the lowered concentration of ferric and cupric ions in solution, respectively. The PM assay is based on the reduction of a Mo (VI)-complex to the corresponding Mo (V)-adduct, without generation of free metal ions in solution.

Finally, the metal-chelating efficacy is of further significance to define the anti-oxidant character of a compound. This ability prevents the participation of transition metal ions, particularly iron and copper, in the generation of free radicals through Fenton’s or Haber-Weiss’ reactions. The transition metal ion chelating capacity of anti-oxidants is evaluated by means of two assays, one measuring the EDTA equivalent iron chelation capacity (EECC) and the other the carnosine equivalent iron chelation capacity (CECC).

It should be considered that experimental results obtained with different methods may sensibly deviate, and the anti-oxidant activity rank have a dissimilar trend, due to many factors including, apart from different chemotypes and mechanisms, light, oxygen, pH, and solvent nature.

Even though, in a number of cases, biological results may be debatable, ongoing investigation on natural agents is encouraged by the well-established link between the oxidative burden and a variety of strongly interconnected human pathologies, including neurodegenerative disorders, metabolic syndrome, cardiovascular diseases, type 2 diabetes (T2D), inflammation, and cancer [11,12]. Several lines of evidence support the role played by oxidative stress in etiology and progression of Alzheimer’s disease (AD) and Parkinson’s disease (PD), characterized by progressive synapse decline and neuronal loss in specific brain areas [13]. Lipid peroxidation, DNA and RNA damage, protein carbonylation, cross-linking, and fragmentation are common oxidative stress-induced hallmarks of AD and PD. Mitochondrial damage consequent to α-amyloid (AD) or α-synuclein (PD) overproduction results in ROS generation, which triggers neuronal injury and apoptosis through disruption of membrane phospholipids and the release of highly reactive malondialdehyde and 4-hydroxy-2,3-nonenal as oxidation by-products and markers, oxidative inactivation of nucleic acid and ATP-related enzymes, and redox imbalance-related increase of oxidized/misfolded proteins [14]. In analogy, oxidative stress is a major risk factor for the development of metabolic diseases. Results from in vitro and in vivo studies suggest that ROS-induced pre-adipocyte proliferation and increase in size of differentiated adipocytes have a causal role in obesity. It has also been disclosed that the selective increase in ROS production in accumulated fat leads to elevation of systemic oxidative stress and is, at least in part, the cause of dysregulation of adipocytokines [15].

One of the most attractive sources of bioactive compounds is represented by peptides: In view of their chemical and structural versatility, along with the intrinsic absence of detrimental effects, they represent ideal molecules to be unveiled as anti-oxidant candidates.

**Figure 1:**
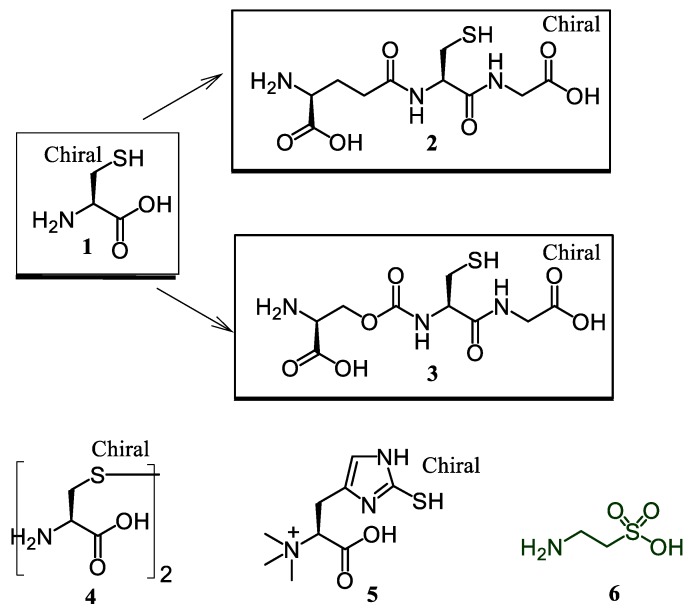
Chemical structures of sulfurated compounds **1**–**6**.

As a prosecution of our ongoing research on bioactive peptides [16,17,18,19], we were interested at first in a comparative in vitro investigation on the anti-oxidant properties of sulfurated amino acids considered either as single units or incorporated in small peptides. Sulfur-containing compounds under study included L-cysteine (Cys) (**1**) with the related thiol-tripeptides, glutathione [H-Glu(Cys-Gly-OH)-OH, GSH] (**2**), and its synthetic gamma-oxa-analogue H-Glo(Cys-Gly-OH)-OH (**3**), and the amino acids, L-cystine (**4**), L-ergothioneine (EGT) (**5**), and taurine (Tau) (**6**) (Figure 1).

**Figure 2:**
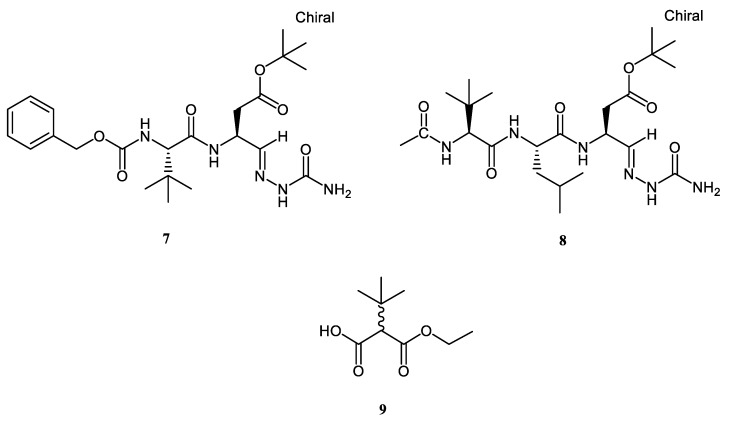
Chemical structures of *t*Leu-based semicarbazones **7** and **8** and *tert*-butyl ethyl malonate **9**.

Additionally, a further set of synthetic compounds, namely Z-*t*Leu-Asp(O*t*Bu)-Sc (**7**), Ac-*t*Leu-Leu-Asp(O*t*Bu)-Sc (**8**), and ethyl 2-(D,L)-*tert*-butyl-*mono*-malonate (**9**), were considered in the same study to assess their unprecedented anti-oxidant properties (Figure 2).

The anti-oxidant profile of compounds **1**–**9** was estimated by means of six complementary in vitro assays (DPPH, ABTS, CUPRAC, FRAP, PM, and EECC). Results are collectively reported in Table 2.

There is accumulating evidence that, in addition to the oxidative damage, the (abnormal) catalytic activity of certain enzymes contribute to the development and progression of neurodegenerative and metabolic disorders; thus, in order to better characterize the protective profile of **1**–**9,** with the hope of disclosing novel multifunctional compounds that can simultaneously modulate various interconnected pathological pathways, we tested their inhibitory effects on the following standard battery of enzymes: Acetylcholinesterase (AChE), butyrylcholinesterase (BChE), tyrosinase, α-glucosidase, and α-amylase. Enzyme inhibition is one of the most fruitful strategies in drug research. The inhibition of key enzymes could alleviate observed symptoms in a variety of pathologies, including Alzheimer’s disease (AD), type 2 diabetes (T2D), obesity, and skin hyperpigmentation [20]. According to the cholinergic hypothesis [21], the approach of inhibiting cholinesterases is widely pursued for the restoration of impaired cholinergic function in AD [22] to improve memory function. Targeting tyrosinase is another frequent strategy thought to be beneficial in the prevention of neuronal degeneration as well as hyperpigmentation problems [23], since the monophenolase activity of this enzyme catalyzes the intermediate conversion of tyrosine in L-DOPA, which is an essential and rate-limiting step in melanin synthesis [24]. Again, considering that α-glucosidase and α-amylase are main enzymes in the carbohydrate catabolism, their inhibition could control blood glucose levels in T2D patients [25,26]. Taken together, the discovery of novel enzyme inhibitors is of great interest to combat the aforementioned diseases in the scientific platform.

The inhibitory activities of our compounds, **1**–**9**, on selected enzymes are compared in Table 3.

## 2. Materials and Methods

### 2.1. Chemistry

Diethyl 2-*tert*-butyl-malonate, glutathione (GSH), amino acids, and their derivatives were purchased from Sigma-Aldrich. All other reagents and solvent were of analytical grade and were supplied from Sigma-Aldrich (Milano, Italy). The synthetic protocol for compound **3** has been previously described [27], as well as the preparative routes to peptides **7** [28] and **8** [17]. ^1^H- and ^13^C-NMR experiments (compound **9**) were recorded on a Varian VXR 300 MHz instrument (δ expressed in ppm). Ethyl 2-(D,L)-tert-butyl-mono-malonate (**9**).

Diethyl 2-*tert*-butyl-malonate (0.71 g, 3.3 mmol) and catalytic amounts of H_2_O (1 mL) were added under stirring to a solution of KOH (0.18 g, 0.3 mmol) in absolute EtOH (7 mL). After 17 h at room temperature, the solvent was evaporated under reduced pressure, the residue taken up in 5% NaHCO_3_, and the resulting aqueous solution washed with AcOEt before acidification by means of 6 N HCl. The aqueous phase (pH = 1) was then extracted with AcOEt and the organic layers washed with H_2_O. Drying and evaporation of the solvent in vacuo gave the expected *mono*-ester **9** as an oil (82%), pure on TLC. *R*_f_ (CHCl_3_/MeOH 99:1) = 0.65; ^1^H-NMR (CDCl_3_): δ 1.12 (s, 9H, *t*Bu CH_3_), 1.28 (d t, ^3^*J* = 7.3 Hz, ^6^*J* = 0.6 Hz, 3H, OCH_2_CH_3_), 3.25 (d, ^6^*J* = 0.6 Hz, 1H, (α)-CH), 4.20 (br q, ^3^*J* = 7.3 Hz, 2H, O*CH*_2_CH_3_), 10.0 (br s, 1H, COOH). ^13^C-NMR (CDCl_3_): δ 14.29 (OCH_2_*CH*_3_), 28.28 (*t*Bu CH_3_), 34.43 (C(α)), 61.28 (O*CH*_2_CH_3_), 61.71 (C(α)), 169.74 (COOR), 173.32 (COOH).

### 2.2. Biological Studies

Antioxidant (DPPH and ABTS radical scavenging, reducing power (CUPRAC and FRAP), phosphomolybdenum and metal chelating (ferrozine method)) and enzyme inhibitory activities (cholinesterase (Elmann’s method), tyrosinase (dopachrome method), α-amylase (iodine/potassium iodide method) and α-glucosidase (chromogenic PNPG method)) were determined using the methods previously described by our published paper [29]. Spectrophotometric measurements for antioxidant and enzyme inhibitory assays were performed with Thermo Scientific Multiskan GO (Thermo Fisher Scientific, Vantaa, Finland).

For the DPPH (1,1-diphenyl-2-picrylhydrazyl) radical scavenging assay: Sample solution (1 mg/mL; 1 mL) was added to 4 mL of a 0.004% methanol solution of DPPH. The sample absorbance was read at 517 nm after a 30 min incubation at room temperature in the dark. DPPH radical scavenging activity was expressed as milligrams of trolox equivalents (mg TE/g sample).

For the ABTS (2,2′-azino-bis(3-ethylbenzothiazoline) 6-sulfonic acid) radical scavenging assay: Briefly, ABTS+ was produced directly by reacting 7 mM ABTS solution with 2.45 mM potassium persulfate and the mixture was allowed to stand for 12–16 h in the dark at room temperature. Prior to beginning the assay, ABTS solution was diluted with methanol to an absorbance of 0.700 ± 0.02 at 734 nm. Sample solution (1 mg/mL; 1 mL) was added to ABTS solution (2 mL) and mixed. The sample absorbance was read at 734 nm after a 30 min incubation at room temperature. The ABTS radical scavenging activity was expressed as milligrams of trolox equivalents (mg TE/g sample).

For the CUPRAC (cupric ion reducing activity) activity assay: Sample solution (1 mg/mL; 0.5 mL) was added to premixed reaction mixture containing CuCl_2_ (1 mL, 10 mM), neocuproine (1 mL, 7.5 mM), and NH_4_Ac buffer (1 mL, 1 M, pH 7.0). Similarly, a blank was prepared by adding sample solution (0.5 mL) to a premixed reaction mixture (3 mL) without CuCl_2_. Then, the sample and blank absorbances were read at 450 nm after a 30 min incubation at room temperature. The absorbance of the blank was subtracted from that of the sample. CUPRAC activity was expressed as milligrams of trolox equivalents (mg TE/g sample).

For the FRAP (ferric reducing antioxidant power) activity assay: Sample solution (1 mg/mL; 0.1 mL) was added to premixed FRAP reagent (2 mL) containing acetate buffer (0.3 M, pH 3.6), 2,4,6-tris (2-pyridyl)-S-triazine (TPTZ) (10 mM) in 40 mM HCl, and ferric chloride (20 mM) in a ratio of 10:1:1 (*v*/*v*/*v*). Then, the sample absorbance was read at 593 nm after a 30 min incubation at room temperature. FRAP activity was expressed as milligrams of trolox equivalents (mg TE/g sample).

For the PM method: Sample solution (1 mg/mL; 0.3 mL) was combined with 3 mL of reagent solution (0.6 M sulfuric acid, 28 mM sodium phosphate, and 4 mM ammonium molybdate). The sample absorbance was read at 695 nm after a 90 min incubation at 95 °C. The total antioxidant capacity was expressed as millimoles of trolox equivalents (mmol TE/g sample).

For the metal chelating activity assay: Briefly, sample solution (1 mg/mL; 2 mL) was added to FeCl_2_ solution (0.05 mL, 2 mM). The reaction was initiated by the addition of 5 mM ferrozine (0.2 mL). Similarly, a blank was prepared by adding sample solution (2 mL) to FeCl_2_ solution (0.05 mL, 2 mM) and water (0.2 mL) without ferrozine. Then, the sample and blank absorbances were read at 562 nm after a 10 min incubation at room temperature. The absorbance of the blank was subtracted from that of the sample. The metal chelating activity was expressed as milligrams of EDTA (disodium edetate) equivalents (mg EDTAE/g sample).

For the cholinesterase (ChE) inhibitory activity assay: Sample solution (1 mg/mL; 50 μL) was mixed with DTNB (5,5-dithio-bis(2-nitrobenzoic) acid (Sigma, St. Louis, MO, USA) (125 μL) and AChE (acetylcholinesterase (Electric ell acetylcholinesterase, Type-VI-S, EC 3.1.1.7, Sigma)), or BChE (butyrylcholinesterase (horse serum butyrylcholinesterase, EC 3.1.1.8, Sigma)) solution (25 μL) in Tris-HCl buffer (pH 8.0) in a 96-well microplate and incubated for 15 min at 25 °C. The reaction was then initiated with the addition of acetylthiocholine iodide (ATCI, Sigma) or butyrylthiocholine chloride (BTCl, Sigma) (25 μL). Similarly, a blank was prepared by adding sample solution to all reaction reagents without enzyme (AChE or BChE) solution. The sample and blank absorbances were read at 405 nm after 10 min incubation at 25 °C. The absorbance of the blank was subtracted from that of the sample and the cholinesterase inhibitory activity was expressed as galantamine equivalents (mgGALAE/g sample).

For the tyrosinase inhibitory activity assay: Sample solution (1 mg/mL; 25 μL) was mixed with tyrosinase solution (40 μL, Sigma) and phosphate buffer (100 μL, pH 6.8) in a 96-well microplate and incubated for 15 min at 25 °C. The reaction was then initiated with the addition of L-DOPA (40 μL, Sigma). Similarly, a blank was prepared by adding sample solution to all reaction reagents without enzyme (tyrosinase) solution. The sample and blank absorbances were read at 492 nm after a 10 min incubation at 25 °C. The absorbance of the blank was subtracted from that of the sample and the tyrosinase inhibitory activity was expressed as kojic acid equivalents (mgKAE/g sample).

For the α-amylase inhibitory activity assay: Sample solution (1 mg/mL; 25 μL) was mixed with α-amylase solution (ex-porcine pancreas, EC 3.2.1.1, Sigma) (50 μL) in phosphate buffer (pH 6.9 with 6 mM sodium chloride) in a 96-well microplate and incubated for 10 min at 37 °C. After pre-incubation, the reaction was initiated with the addition of starch solution (50 μL, 0.05%). Similarly, a blank was prepared by adding sample solution to all reaction reagents without enzyme (α-amylase) solution. The reaction mixture was incubated for 10 min at 37 °C. The reaction was then stopped with the addition of HCl (25 μL, 1 M). This was followed by addition of the iodine-potassium iodide solution (100 μL). The sample and blank absorbances were read at 630 nm. The absorbance of the blank was subtracted from that of the sample and the α-amylase inhibitory activity was expressed as acarbose equivalents (mmol ACE/g sample).

For the α-glucosidase inhibitory activity assay: Sample solution (1 mg/mL; 50 μL) was mixed with glutathione (50 μL) and α-glucosidase solution (from *Saccharomyces cerevisiae*, EC 3.2.1.20, Sigma) (50 μL) in phosphate buffer (pH 6.8) and PNPG (4-nitro-phenyl- α-D-glucopyranoside, Sigma) (50 μL) in a 96-well microplate and incubated for 15 min at 37 °C. Similarly, a blank was prepared by adding sample solution to all reaction reagents without enzyme (α-glucosidase) solution. The reaction was then stopped with the addition of sodium carbonate (50 μL, 0.2 M). The sample and blank absorbances were read at 400 nm. The absorbance of the blank was subtracted from that of the sample and the α-glucosidase inhibitory activity was expressed as acarbose equivalents (mmol ACE/g sample). One-way analysis of variance (ANOVA) was done to determine any differences between the tested samples following a Tukey’s test. *p* < 0.05 were assigned to be statistically significant. The statistical procedures were performed by SPPS v. 17.0.

## 3. Results and Discussion

Compounds **1**–**6** are representative of a different oxidation status of the sulfur atom, and their electron-donating character varies accordingly. Compounds **1**–**3** contain a sulfhydryl group, which is strongly nucleophilic and may act as a radical quencher, reductant, and metal chelator. GSH (**2**) is the most abundant intracellular thiol in mammals, and its role as a detoxifying agent is not questionable. In its anti-oxidant behavior, the natural tripeptide was compared to analogue **3**, containing a stable OCONH bond as the CONH surrogate at the (α)-glutamyl junction, which has been previously reported in the course of our previous studies on GSH chemical modification [27,30]. The protective effects of EGT (**5**) against the oxidative damage, both in vitro and in vivo, have been widely documented, despite some conflicting results [31]. This natural betaine derivative is characterized by a distinctive thione/thiol tautomeric function. L-cystine (**4**), presenting a disulfide bridge, is prone to oxidative demolition to form sulfenic, sulfinic, and sulfonic species. The last compound in the series, taurine (**6**), contains a completely oxidized sulfur atom. Table 2 summarizes the efficiency data for compounds **1**–**9** in terms of their free radical scavenging ability (ABTS and DPPH), reducing power (CUPRAC, FRAP, and PM), and metal chelating activity.

In the group of sulfurated compounds analyzed in this study, **1**–**5** present an excellent anti-oxidant profile. In detail, compounds **1**–**5** exhibited good DPPH scavenging activities, with well-aligned values ranging from 106.07 ± 0.57 mg TE/g sample, for the glutathione oxa-analogue **3**, to 65.48 ± 2.81 57 mg TE/g sample, for GSH (**2**), whilst taurine (**6**) was found devoid of activity. The radical scavenging activity was found to decrease in the order: H-Glo(Cys-Gly-OH)-OH (**3**) ≥ L-cysteine (**1**) ≥ L-cystine (**4**) > EGT (**5**) ≥ GSH (**2**).

In the ABTS assay, L-cysteine (**1**), GSH (**2**), and EGT (**5**) showed the highest radical cation scavenging ability (245.14 ± 0.21 mg TE/g sample, 196.89 ± 0.38 mg TE/g sample, and 123.88 ± 0.24 mg TE/g sample, respectively), compared to compounds **3**, **4**, and **6**, which do not trap ABTS**^•+^** effectively.

Quite notable results were obtained also in the CUPRAC reducing power assay conducted on **1**–**6**, with the strongest activity shown by L-cysteine (**1**) (939.97 ± 2.96 mg TE/g sample), followed by, in descending order, EGT (**5**) (534.55 ± 7.87 mg TE/g sample), GSH (**2**) (389.74 ± 7.88 mg TE/g sample), L-cystine (**4**) (124.72 ± 4.13 mg TE/g sample), and the isosteric glutathione **3** (69.36 ± 0.97 mg TE/g sample). Taurine (**6**) showed the lowest activity in the assay, presenting a value of 26.19 ± 0.53 mg TE/g sample.

A similar trend was observed for the sulfurated compounds in the FRAP rank, with their Fe^3+^ reducing activities descending in the following order **1** >> **2** > **5** > **4** ≈ **3** > **6**. A significant value was registered for L-cysteine (**1**) (761.90 ± 16.69 mg TE/g sample), resulting 5-, 6-, and even 18-fold higher than the corresponding parameters of GSH (**2**) (143.03 ± 6.68 mg TE/g sample), EGT (**5**) (114.94 ± 1.75 mg TE/g sample), and the lined-up couple, L-cystine (**4**) (41.59 ± 0.35 mg TE/g sample) and H-Glo(Cys-Gly-OH)-OH (**3**) (41.44 ± 0.86 mg TE/g sample), respectively.

In the PM assay, the most efficient compounds were, in descending order, GSH (**2**) (2.50 ± 0.11 mmol TE/g sample), EGT (**5**) (2.46 ± 0.02 mmol TE/g sample), and L-cysteine (**1**) (2.28 ± 0.02 mmol TE/g sample), while the remaining **3**, **4**, and **6** exhibited only modest or negligible activity.

Finally, L-cystine (**4**) and H-Glo(Cys-Gly-OH)-OH (**3**) are the only sulfur-containing compounds to reveal a consistent and comparable metal chelating activity (84.77 ± 0.11 mg EDTAE/g sample and 70.91 ± 0.17 mg EDTAE/g sample, respectively), followed by taurine (**6**) with a ca. 4-fold decrease in efficacy (20.45 ± 2.08 mg EDTAE/g sample).

The analysis of the data concerning the sulfurated compounds **1**–**5** reveals a strong correlation between the ABTS and FRAP assays. With respect to the comparison between the ferric reducing potential, determined by the FRAP assay, and the metal chelating activity, an interesting behavior can be observed for the two thiol molecules, L-cystine (**4**) and H-Glo(Cys-Gly-OH)-OH (**3**): They show in fact a Fe^3+^ reducing power in the low-medium range compared to the more active compounds of **1**, **2** and **5**. This effect may be due to the strong metal chelating activity of **3** and **4**, not shared by the other compounds in the series, which interfere with the metal ion-containing FRAP assay [32]. It is interesting to note that, with respect to CUPRAC, FRAP values are subjected to larger variations, which can be explained with the iron’s slower reaction kinetics if compared to copper, and the reported interferences, occurring particularly with thiols [32].

In our experiments, L-cysteine (**1**) revealed the strongest free radical scavenging power and metal reducing capacity; it is worth noting that the two Cys-based tripeptides, GSH (**2**) and its oxa-analogue **3**, although optimally ranked, were less efficient, thus suggesting that the anti-oxidant potential of the sulfydrylated amino acid is negatively affected when it is inserted in (α)-glutamyl peptides.

The established potential of the natural anti-oxidant EGT (**5**) has been confirmed by our results; however, in contrast with previous studies observing the formation of stable bivalent metal complexes through the SH group, we did not observe a significant metal chelating activity for this thiolate [33]. L-cystine (**4**) showed a somewhat less relevant activity with respect to **5**. This finding is in agreement with the medium anti-oxidant character of the disulfide unit, which is still able to undergo further oxidation.

As outlined in Table 2, taurine (**6**) displayed none or very weak activity in DPPH, ABTS, FRAP, and PM assays, manifesting, however, moderate CUPRAC and metal chelating power. The activity of taurine towards free radicals has not been demonstrated incontrovertibly. Although the exact mechanisms underlying the low free radical scavenging effect of this amino acid remain to be established, it has been suggested that it might be due to the lack of a readily oxidizable functional group [34].

Regarding non-sulfurated compounds, both peptides **7** and **8** are semicarbazone derivatives, and incorporate the non-proteinogenic amino acid, *tert*-leucine (*t*Leu). Ongoing attention is focused on the chemical, conformational, and medicinal aspects of this natural aliphatic residue [35,36,37]. Due to its (α)-branched *tert*-butyl side chain, *t*Leu is strongly lipophilic and much more bulky with regards to isomeric leucine and isoleucine. Excellent reports have highlighted the role of this amino acid, in terms of steric and polar effects, in decreasing peptide radical formation and stabilization in vivo [38]. On the other hand, semicarbazones, as well as thiosemicarbazones and hydrazones, are known to possess a wide array of biological activities, essentially due to their ability to form strongly H-bond stabilized chelates with heavy metals [39].

The results of the same panel of tests conducted on Z-*t*Leu-Asp(O*t*Bu)-Sc (**7**), Ac-*t*Leu-Leu-Asp(O*t*Bu)-Sc (**8**), and ethyl 2-(D,L)-*tert*-butyl-*mono*-malonate (**9**) indicated that this set of compounds possess a moderate anti-oxidant character in comparison with the sulfurated ones (Table 2). They were not active in the DPPH assay, and exhibited extremely large variation in their reducing power and metal chelating capacity. We hypothesized that, owing to steric inaccessibility of their *t*Bu-scaffolds, **7**–**9** may react very slow or even be inert to DPPH. However, Ac-*t*Leu-Leu-Asp(O*t*Bu)-Sc (**8**) showed a complete anti-oxidant profile, with a remarkable activity in ABTS (48.01 ± 1.41 mg TE/g sample), CUPRAC (81.31 ± 1.85 mg TE/g sample), FRAP (170.47 ± 5.50 mg TE/g sample), PM (2.62 ± 0.13 mmol TE/g sample), and metal chelating power (82.70 ± 3.01 mg EDTAE/g sample) assays. This latter parameter is perfectly in line with the values displayed by the most active chelating agents between the sulfur-containing compounds. Both Z-*t*Leu-Asp(O*t*Bu)-Sc (**7**) and 2-(D,L)-*tert*-butyl-*mono*-malonate (**9**) were found devoid of radical scavenging activity; however, Z-*t*Leu-Asp(O*t*Bu)-Sc (**7**) is equipotent to Ac-*t*Leu-Leu-Asp(O*t*Bu)-Sc (**8**) in regard to Cu^2+^ reducing power (84.30 ± 1.39 mg TE/g sample), and its metal chelating activity is still notable, resulting in half the value of the tripeptide (43.69 ± 3.40 mg EDTAE/g sample), although it was less efficient in the FRAP and PM assays. As far as 2-(D,L)-*tert*-butyl-*mono*-malonate (**9**) is concerned, its metal reducing and chelating behavior is modest and very similar to the one exhibited by taurine (**6**). In conclusion, Z-*t*Leu-Asp(O*t*Bu)-Sc (**7**) and Ac-*t*Leu-Leu-Asp(O*t*Bu)-Sc (**8**), the first *t*Leu-containing peptides to be tested in vitro for their anti-oxidant potential, disclosed quite interesting properties. The overall behavior of tripeptide **8** is superior compared to dipeptide **7**.

Based on these encouraging data, and for the purpose of obtaining further insights on the protective role of this set of compounds, we investigated **1**–**9** as inhibitors of acetylcholinesterase (AChE), butyrylcholinesterase (BChE), tyrosinase, α-glucosidase, and α-amylase. The inhibitory activity results, collected in Table 3, revealed that our compounds are all effective as tyrosinase inhibitors. In particular, L-cysteine (**1**), H-Glo(Cys-Gly-OH)-OH (**3**), and L-cystine (**4**) presented an impressive and quite comparable inhibition profile against the enzyme, with activity values of 216.40 ± 0.17 mg KAE/g sample, 216.95 ± 0.17 mg KAE/g sample, and 217.16 ± 0.55 mg KAE/g sample, respectively. A marked inhibitory effect was evidenced for peptides, Z-*t*Leu-Asp(O*t*Bu)-Sc (**7**) and Ac-*t*Leu-Leu-Asp(O*t*Bu)-Sc (**8**) (163.87 ± 0.90 mg KAE/g sample and 152.69 ± 1.19 mg KAE/g sample, respectively). The corresponding parameter for EGT (**5**) resulted ca. half the value of **4** (100.97 ± 1.71 mg KAE/g sample), while GSH (**2**), ethyl 2-(D,L)-*tert*-butyl-*mono*-malonate (**9**), and taurine (**6**) displayed an almost 5-/6-fold decrease in inhibitory activity with regards to the most active compound (45.60 ± 0.15 mg KAE/g sample 45.37 ± 0.52 mg KAE/g sample, and 33.87 ± 1.59 mg KAE/g sample, respectively). All compounds were practically inactive towards the other selected enzymes.

## 4. Conclusions

The biological relevance of amino acids and peptides as anti-oxidants and protective agents for human health needs to be explored. They offer the advantages of being non-toxic, potent, and chemically versatile substances, with generally good pharmacokinetics and well-defined metabolic destiny. In this paper, amino acids and peptides were tested in vitro for the first time to assess their anti-oxidant potential and inhibitory activity towards a panel of enzymes involved in the pathogenesis of relevant neurodegenerative and metabolic disorders, including AD and T2D.

Sulfur-containing compounds, **1**–**5**, displayed the best anti-oxidant character in the series. Synthetic peptides, **7** and **8**, characterized by the presence of the non-coded *t*Leu residue in their sequence and derivatized as semicarbazones, showed good metal reducing power and strong metal chelating activity.

Furthermore, an inhibitory effect on tyrosinase activity was observed for all the evaluated compounds. Taken together, our in vitro results demonstrated that the compounds under study could help in reducing the-enzyme-induced toxicity associated with oxidative stress involved in the progression of neurodegenerative and metabolic diseases. Closer investigations will be necessary to unravel the multifaceted potential in the bioactivity of these compounds.

## Figures and Tables

**Table 1:** In vitro anti-oxidant capacity assays used for assessing the total anti-oxidant capacity (TAC) of compounds **1**–**9**.

Mechanisms of Assays	Type of Assay	pH	Solvent	Anti-Oxidant Character
Assays involving Single Electron Transfer (SET) reactions ● Non-competitive reduction-based assays	FRAP (Ferric ion Reducing Anti-oxidant Power) ● The assay does not sufficiently responds to thiols due to the kinetic inertness of high-spin Fe(III) in the TPTZ complex	acid	water	hydrophilic
	CUPRAC (CUPric Reducing Anti-oxidant Capacity) ● The reagent rapidly oxidizes thiol-containing anti-oxidants	neutral	alcohols, acetone, DCM, alcohol-water mixtures	lipophilic and hydrophilic
	PM (Phosphomolybdenum assay)	4–5	water	hydrophilic
Assays involving mixed-mode Hydrogen Atom Transfer (HAT) /SET reactions ● Non-competitive scavenging and reduction-based assays	DPPH (2,2-Di-Phenyl-1-Picryl-Hydrazyl)	3–7.5	methanol, ethanol alcohol-water mixtures	lipophilic and hydrophilic
	ABTS (2,2-Azino-Bis(3-ethylbenzo-Thiazoline)-6-Sulphonic acid ● Thiols are oxidized by ABTS^.+^ radical cation to higher oxidation levels (sulfenic and sulfinic acids)	acid	water, ethanol	lipophilic and hydrophilic

**Table 2:** Antioxidant capacity evaluation for compounds **1**–**9**.

Compounds	DPPH (mgTE/g Sample)	ABTS (mgTE/g Sample)	CUPRAC (mgTE/g Sample)	FRAP (mgTE/g Sample)	Phosphomolybdenum (mmolTE/g Sample)	Metal Chelating (mgEDTAE/g Sample)
L-cysteine (**1**)	102.50 ± 1.43 ^a^	245.14 ± 0.21 ^a^	939.97 ± 2.96 ^a^	761.90 ± 16.69 ^a^	2.28 ± 0.02 ^b^	not active
GSH (**2**)	65.48 ± 2.81 ^b^	196.89 ± 0.38 ^b^	389.74 ± 7.88 ^c^	143.03 ± 6.68 ^c^	2.50 ± 0.11 ^a^	not active
H-Glo(Cys-Gly-OH)-OH (**3**)	106.07 ± 0.54 ^a^	not active	69.36 ± 0.97 ^f^	41.44 ± 0.86 ^c^	0.21 ± 0.04 ^e^	70.91 ± 0.17 ^b^
L-cystine (**4**)	100.79 ± 0.40 ^a^	1.20 ± 0.08 ^f^	124.72 ± 4.13 ^d^	41.59 ± 0.35 ^e^	0.11 ± 0.01 ^f^	84.77 ± 0.11 ^a^
Ergothioneine (**5**)	73.23 ± 0.13 ^c^	123.88 ± 0.24 ^c^	534.55 ± 7.87 ^b^	114.94 ± 1.75 ^d^	2.46 ± 0.02 ^a^	1.35 ± 0.27 ^f^
Taurine (**6**)	not active	5.47 ± 0.14 ^e^	26.19 ± 0.53 ^g^	13.47 ± 0.63 ^a^	0.05 ± 0.01 ^g^	20.45 ± 2.08 ^d^
Z-*t*leu-Asp (O*t*Bu)-Sc (**7**)	not active	not active	84.30 ± 1.39 ^e^	30.33 ± 1.44 ^f^	0.36 ± 0.01 ^d^	43.69 ± 3.40 ^c^
Ac-*t*Leu-Leu-Asp(O*t*Bu)-Sc (**8**)	not active	48.01 ± 1.41 ^d^	81.31 ± 1.85 ^e^	170.47 ± 5.50 ^b^	2.62 ± 0.13 ^a^	82.70 ± 3.01 ^a^
Ethyl 2-*t*Bu-(DL)-mono-malonate (**9**)	not active	not active	27.41 ± 3.53 ^g^	14.29 ± 0.36 ^g^	0.54 ± 0.01 ^c^	13.76 ± 3.27 ^e^

TE, Trolox equivalents; EDTAE, Ethylenediaminetetraacetic acid equivalents; different letters indicate differences in the tested samples (*p* < 0.05).

**Table 3:** In vitro enzymatic assays for compounds **1**–**9.**

Compounds	AChE Inhibition (mgGALAE/g)	BChE Inhibition (mgGALAE/g)	Tyrosinase Inhibition (mgKAE/g)	Amylase Inhibition (mmolACAE/g)	Glucosidase Inhibition (mmolACAE/g Sample)
L-cysteine (**1**)	5.12 ± 0.15 ^a^	6.14 ± 0.08 ^ab^	216.40 ± 0.17 ^a^	0.06 ± 0.01 ^e^	not active
GSH (**2**)	3.62 ± 0.04 ^b^	6.13 ± 0.12 ^ab^	45.60 ± 0.15 ^e^	0.03 ± 0.01 ^f^	not active
H-Glo(Cys-Gly-OH)-OH (**3**)	not active	6.39 ± 0.01 ^a^	216.95 ± 0.17 ^a^	1.37 ± 0.03 ^b^	1.69 ± 0.01 ^b^
L-cystine (**4**)	5.24 ± 0.02 ^a^	6.38 ± 0.01 ^a^	217.16 ± 0.55 ^a^	1.95 ± 0.04 ^a^	1.68 ± 0.02 ^b^
Ergothioneine (**5**)	3.01 ± 0.01 ^c^	0.18 ± 0.05 ^d^	100.97 ± 1.71 ^d^	0.07 ± 0.01 ^e^	not active
Taurine (**6**)	0.45 ± 0.03 ^e^	0.07 ± 0.01 ^e^	33.87 ± 1.59 ^f^	0.07 ± 0.01 ^e^	3.96 ± 0.95 ^a^
Z-*t*leu-Asp (O*t*Bu)-Sc (**7**)	not active	6.37 ± 0.01 ^a^	163.87 ± 0.90 ^b^	0.31 ± 0.06 ^c^	1.69 ± 0.01 ^b^
Ac-*t*Leu-Leu-Asp(O*t*Bu)-Sc (**8**)	5.17 ± 0.07 ^a^	6.33 ± 0.01 ^a^	152.69 ± 1.19 ^c^	0.39 ± 0.08 ^c^	not active
ethyl 2-*t*Bu-(DL)-mono-malonate (**9**)	2.61 ± 0.01 ^d^	2.96 ± 0.08 ^c^	45.37 ± 0.52 ^e^	0.20 ± 0.02 ^d^	0.84 ± 0.01 ^c^

GALAE, Galatamine equivalents; KAE, Kojic acid equivalents; ACAE, Acarbose equivalents; different letters indicate differences in the tested samples (*p* < 0.05).

## Abbreviations

ABTS	2,2′-azino-bis(3-ethylbenzothiazoline-6-sulphonic acid)
ACE	Acarbose equivalents
AChE	acetylcholinesterase
AcOEt	Ethyl acetate
AD	Alzheimer’s disease
ATCI	Acetylthiocholine iodide
BChE	Butyrylcholinesterase
BTCI	Butyrylthiocholine chloride
CECC	Carnosine Equivalent Iron Chelation Capacity
ChE	Cholinesterase
^13^C-NMR	Carbon-13 nuclear magnetic resonance
CUPRAC	Cupric ion reducing antioxidant capacity
DPPH	2,2-diphenyl-1-picrylhydrazyl
DTNB	5,5-dithio-bis(2-nitrobenzoic acid)
EDTA	Ethylenediaminetetraacetic Acid
EECC	EDTA Equivalent Iron Chelation Capacity
EGT	L-ergothioneine
EtOH	Ethanol
FRAP	Ferric reducing ability of plasma
GALAE	Galantamine equivalents
GSH	Gluthatione
HAT	Hydrogen atom transfer
^1^H-NMR	Proton nuclear magnetic resonance
KAE	Kojic acid
L-DOPA	Levo-dihydroxy-phenylalanine
LPA	Lipid peroxidation assay
NEAC	Non enzymatic antioxidant capacity
ORAC	Oxygen radical absorbance capacity
OS	Oxidative strees
PM	Phosphomolybdenum
PNPG	p-nitrophenol-alfa-D-glucopyranoside
RNS	Reactive nitrogen species
ROS	Reactive Oxygen Species
SET	Single electron transfer
TAC	Total anti-oxidant capacity
Tau	Taurine
T2D	type 2 diabetes
TEAC	Trolox equivalent antioxidant capacity
TLC	Thin layer chromatography
TPTZ	2,4,6-tris(2-pyridyl)-S-triazine
TRAP	Total radical trapping antioxidant parameter
